# Expression and significance of mammalian target of rapamycin in cutaneous squamous cell carcinoma and precancerous lesions

**DOI:** 10.1080/21655979.2021.1984719

**Published:** 2021-12-07

**Authors:** Gongjun Xu, Jinxian Fang, Jinlun Xu, Zhen Shen, Chiqing Huang, Yixiu Jiang

**Affiliations:** Department of Dermatology,Jinhua Fifth Hospital, JinHua, ZheJiang,China

**Keywords:** Mtor, squamous cell, carcinoma, precancerous lesion, skin

## Abstract

The aim of this study is to explore the role of mammalian target of rapamycin (mTOR) in cutaneous squamous cell carcinoma (CSCC), Bowen’s disease (BD), and actinic keratosis (AK) with squamous cell differentiation abnormality and its relationship with the degree of tumor proliferation. Thirty cases of clinical paraffin specimens of CSCC, BD, and AK were each collected from Jinhua Fifth Hospital, while 30 cases of normal skin specimens surgically resected in Department of Plastic Surgery were selected as controls. The expressions of mTOR and Ki-67 in tissues were detected by immunohistochemical staining. The positive expression rate of mTOR in the CSCC group was higher than those in the BD group and AK group (*P* < 0.05), while it was higher in the BD group and AK group than in the normal skin group (*P* < 0.05). The CSCC group had a higher positive expression rate of Ki-67 than the AK group (*P* < 0.01). The results of logistic regression analysis showed that the pathogenic site [odds ratio (OR) = 1.189, 95% confidence interval (95%CI): 1.028–1.381, *P* = 0.021], course of disease (OR = 2.059, 95%CI: 1.036–4.087, *P* = 0.043), and differentiation degree (OR = 1.325, 95%CI: 1.169–1.512, *P* = 0.001) were independent factors for the positive expression of mTOR. OR>1, indicating that the factor is a risk factor. The expression levels of mTOR in CSCC, BD, and AK were positively correlated with the expression level of Ki-67 (*r* = 0.827, *P* < 0.01, *r* = 0.608, *P* < 0.01, *r* = 0.368, *P* = 0.045). These results suggest that mTOR may be involved in the pathogenesis of CSCC, and related to the proliferation degree of CSCC, as an index reflecting the proliferation status of CSCC.

## INTORDUCTION

Cutaneous squamous cell carcinoma (CSCC), a malignant tumor originating from the epidermis or adnexal keratinocytes frequently, occurs in middle-aged and elderly people. The number of confirmed cases of CSCC is increasing at a rate of 2.6% every year in China. Bowen’s disease (BD) is *in situ* CSCC, which can be primary or occur based on actinic keratosis (AK). Approximately 3–5%, some argue up to 20–30%, of BD can progress into invasive squamous cell carcinoma. AK, also known as solar keratosis, is a kind of precancerous lesion caused by the damage of long-term sun exposure to the skin, and its incidence rate is 0.006–0.7%, which increases in areas with strong ultraviolet radiation. At the same time, it has been found that 0–0.075% of AK is transformed into CSCC every year [1].

Recent studies have shown that the occurrence of CSCC has a relationship with the abnormalities of multiple oncogenes and cancer suppressor genes. At the same time, the role of molecular signal transduction pathways in CSCC has attracted increasingly more attention. Among various molecular signal transduction pathways, the mammalian target of rapamycin (mTOR) signal transduction pathway closely related to cell proliferation has become a hot spot of domestic and international research. MTOR has been confirmed to be closely associated with the occurrence of various tumors, which is a new direction for targeted cancer therapy [2]. Ki-67, an essential enzyme for DNA replication and synthesis into mRNA, can reflect the cell proliferative activity and cycle more accurately, which is currently the most widely used index for assessing the cell proliferation status. Measuring Ki-67 is a reliable and simple method to evaluate the proliferation level of various human tumor tissues. This paper hypothesizes that the activation of the mTOR signaling pathway promotes the occurrence and proliferation of CSCC. Therefore, in the present study, the expressions of mTOR and Ki-67 were detected in CSCC, BD, and AK tissues, the correlation between the expressions of mTOR and Ki-67 was analyzed, and the role of mTOR in the above three kinds of skin diseases with squamous cell differentiation abnormality and its relationship with the degree of tumor proliferation were explored, so as to provide new ideas for the diagnosis and treatment of CSCC.

## SUBJECTS AND METHODS

### Subjects

Upon the approval by the Medical Ethics Committee of our hospital, the specimens were collected from CSCC, BD and AK patients undergoing operation in Department of Dermatology from January 2015 to December 2018. CSCC were primarily caused by cumulative UV exposure and were usually found in areas that were often exposed to sunlight (the edges of the ears, lower lip, face, bald scalp, neck, hands, arms, and legs). All patients were diagnosed and had complete clinical data, and none of them received special treatments such as laser therapy, photodynamic therapy, radiotherapy, and chemotherapy before operation. They were divided into CSCC group (n = 30), BD group (n = 30), and AK group (n = 30). Meanwhile, 30 cases of normal skin specimens surgically resected in Department of Plastic Surgery were selected as controls.

## Methods

### General data were collected

General data such as the patient’s age (years), gender (male and female), and course of disease (from the time of symptom onset to the time of treatment) were collected.

### Specimen treatment

Following surgical resection, the specimens were immediately rinsed with normal saline, trimmed, fixed with 10% formalin for 24 h, embedded in paraffin, and serially sectioned at a thickness of 4 *μ*m.

### Immunohistochemical staining

Rabbit antihuman mTOR polyclonal antibody (1:400, bs-1992 R, Beijing BIOSS Antibodies Co., Ltd.) and rabbit antihuman Ki-67 polyclonal antibody (1:400, bs-2130 R, Beijing BIOSS Antibodies Co., Ltd.) were used as primary antibodies. Streptavidin-peroxidase (SP) immunohistochemical staining was performed according to the instructions of kits. Staining steps [3]: Paraffin sections were deparaffinized to water and incubated with 3% H_2_O_2_ at 26°C for 5–10 min to eliminate endogenous peroxidase activity. The sections were rinsed with distilled water and soaked in diluted Phosphate Buffer Saline (PBS) for 5 min. The sections were blocked with 5–10% normal goat serum (PBS) and incubated at 26°C for 10 min. Pour out the serum and do not wash. The sections were dripped with the primary antibody (rabbit anti-human mTOR polyclonal antibody, rabbit anti-human Ki-67 polyclonal antibody) diluted in appropriate proportions, incubated at 37°C for 1–2 h or 4°C for 12 h, and rinse with PBS, 5 min × 3 times. The sections were dripped with a biotin-labeled secondary antibody diluted in an appropriate ratio (1% Bovine Serum Albumin-PBS dilution), incubated at 37°C for 10–30 min, and rinse with PBS, 5 min × 3 times. The sections were dripped with horseradish enzyme-labeled streptavidin diluted in PBS, incubated at 37°C for 10–30 min, and rinse with PBS, 5 min × 3 times. Diaminobenzidine (DAB) chromogenic agent develops color. Rinse thoroughly with tap water, counterstain with hematoxylin, and mount the film. Tissues known to contain the antigens to be tested (mTOR and Ki-67) were used as positive controls. PBS instead of primary antibody was used as negative controls.

### Determination criteria for immunohistochemical staining results

The films were read by two dermatological pathologists (Huang Chiqing and Jiang Yixiu), and the results were determined after discussion. The yellow or brown yellow particles in the cytoplasm indicated the positive staining of mTOR. Ki-67 was mainly expressed in the proliferating cell nucleus, the negative nucleus was light blue in the microscopic section, and the brown or dark brown particles in the nucleus indicated the positive staining of Ki-67. In 5 nonrepeated fields randomly selected under a microscope (×400), a comprehensive score (H-scores) was given based on the number of positive cells and the intensity of staining. The scoring criteria used in this study were modified based on the criteria reported in a previous literature [4] as follows: no positive cells: 0 points, 1%≤ positive cells ≤25%: 1 point, 25%< positive cells ≤50%: 2 points, 50%< positive cells ≤75%: 3 points, and positive cells >75%: 4 points. The stain intensity (H-scores) was scored as follows: 0, negative (no staining); 1, week (yellow); 2 moderate (brown yellow); and 3, strong (dark brown). For Ki-67 staining sections, the stain intensity (H-scores) was scored as follows: 0, negative (light blue); 1, week (light brown); 2 moderate (brown); and 3 strong (dark brown). The total score was calculated with the product of the two. Then, the total score of 0–1 point is for negative (-), 2–3 points for weakly positive (+), 4–6 points for moderately positive (++), and >6 points for strongly positive (+++). Finally, the total score <4 points indicated negative (-) and that ≥4 points indicated positive (+) for the results of immunohistochemical staining of tissue specimens.

### Statistical analysis

SPSS24.0 software was used for data management and statistical analysis. The qualitative data were described by n(%). The intergroup comparison of qualitative data was conducted using the χ^2^ test or Fisher’s exact probability test, and partition of chi-square was used for pairwise comparison [5]. The quantitative data were expressed by mean ±SD. The expression levels of mTOR and Ki-67 in the four groups were subjected to the Kruskal-Wallis *H* test and compared between two groups through the Nemenyi test [6]. In multivariate analysis, hierarchical logistic regression was performed [7], with mTOR expression as the binary dependent variable and the binary variables in univariate analysis as independent variables. Spearman’s correlation analysis [8] was conducted for the expressions of mTOR and Ki-67. *P* < 0.05 was considered statistically significant.

## RESULTS

By detecting the expression of mTOR and Ki-67 in CSCC, BD, and AK tissues, the correlation between the expression of mTOR and Ki-67 and the correlation between the positive expression of mTOR and the clinicopathological features of CSCC were analyzed, so as to provide new ideas for the diagnosis and treatment of CSCC.

### General data

There were 30 cases in the CSCC group, including 13 males and 17 females, the mean age was 67.75 ± 7.85 years (range, 43–88 years), and the course of disease ranged from two months to 10 years. There were 30 cases in the BD group, including 18 males and 12 females, the mean age was 69.61 ± 7.32 years (range, 47–88 years), and the course of disease ranged from 10 months to 30 years. There were 30 cases in the AK group, including eight males and 22 females, the mean age was 71.23 ± 6.71 years (range, 51–87 years), and the course of disease ranged from 1 month to 10 years. In the normal skin group, 14 males and 16 females, with the mean age of 63.37 ± 5.49 years (range, 38–82 years), were enrolled. No statistically significant differences were found in the age and gender composition among the four groups.

### Expressions of mTOR in CSCC, BD, AK, and normal skin

**Figure 1.**
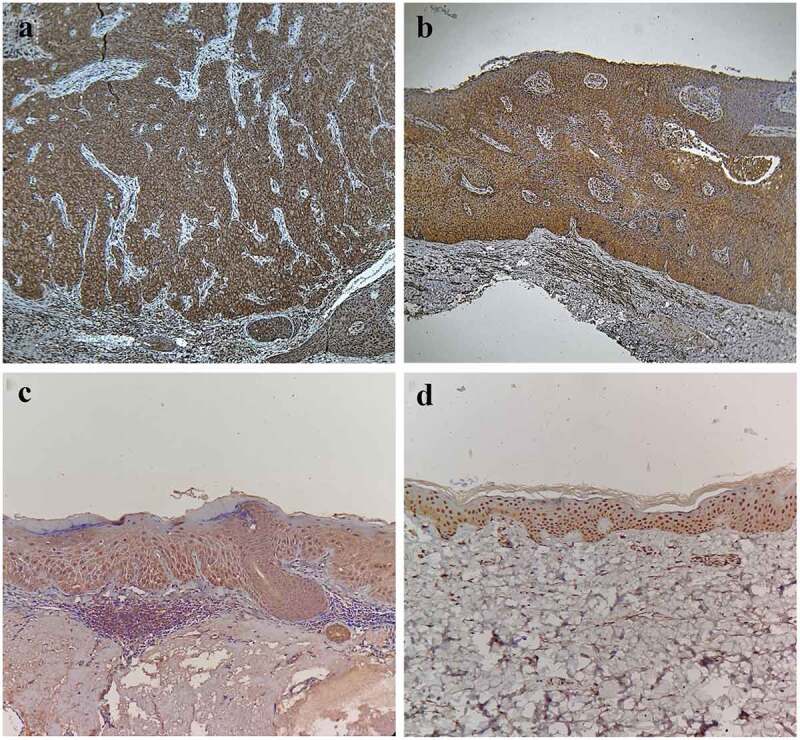
Expressions of mTOR in CSCC, BD, AK, and normal skin. (a) Positive expression of mTOR in CSCC (SP, ×200), Positive(+++). (b) Positive expression of mTOR in BD (SP, ×200), Positive(++). (c) Positive expression of mTOR in AK (SP, ×200), Positive(++). (d) Positive expression of mTOR in normal skin (SP, ×200), Positive(++)

In the CSCC group, there were a total of 22 cases (73.33%) of positive expression (Figure 1a), dominated by strong positive expression. In the BD group, there were a total of 14 cases (46.67%) of positive expression (Figure 1b), dominated by moderate positive expression. In the AK group, there were a total 11 cases (36.67%) of positive expression (Figure 1c), dominated by moderate positive expression. In the normal skin group, positive expression was found in four cases (13.33%) (Figure 1d), including three cases of moderate positive expression and one case of strong positive expression. The case with strong positive expression of normal skin was 46 years old male.

It can be seen that the positive expression rate of mTOR had statistically significant differences among the four groups (χ^2^ = 6.71, *P* < 0.05). Furthermore, it was found through partition of chi-square that the positive expression rate of mTOR in the CSCC group was higher than that in the BD group and AK group (χ^2^ = 4.44, *P* < 0.05; χ^2^ = 8.15, *P* < 0.01), while it was higher in the BD group and AK group than that in the normal skin group (χ^2^ = 7.94, *P* < 0.01; χ^2^ = 4.36, *P* < 0.05). There was no statistically significant difference in the positive expression rate of mTOR between the BD group and the AK group (χ^2^ = 0.62, *P* > 0.05). Besides, the mTOR expression had statistically significant differences among the four groups in the Kruskal-Wallis *H* test (*H* = 58.09, *P* < 0.05). The results of pairwise comparison *via* the Nemenyi test revealed that the difference in the mTOR expression was statistically significant in the CSCC group compared with the BD group, AK group, and normal skin group (*P* < 0.05), and the same was true in he BD group and AK group compared with the normal skin group (*P* < 0.05), but it was not statistically significant between the BD group and AK group (*P* > 0.05), as shown in Table 1.

**Table 1.** Expressions of mTOR and Ki-67in CSCC, BD, AK, and normal skin [n (%)]

	mTOR							Ki-67				
Group	n	-	+	++	+++	Positive [n (%)]		-	+	++	+++	Positive [n (%)]
CSCC	30	2	6	8	14	22(73.33)		0	2	12	16	28(93.33)
BD	30	4	12	8	6	14(46.67)		2	5	10	13	23(76.67)
AK	30	8	11	8	3	11(36.67)		4	8	11	7	18(60.00)
Normal skin	30	17	9	3	1	4(13.33)		0	0	0	0	0(0.00)
χ^2^		6.71						5.57				
*H*		58.09						33.87				
*P*		<0.05						<0.05				

### Expressions of Ki-67 in CSCC, BD, AK, and normal skin

**Figure 2.**
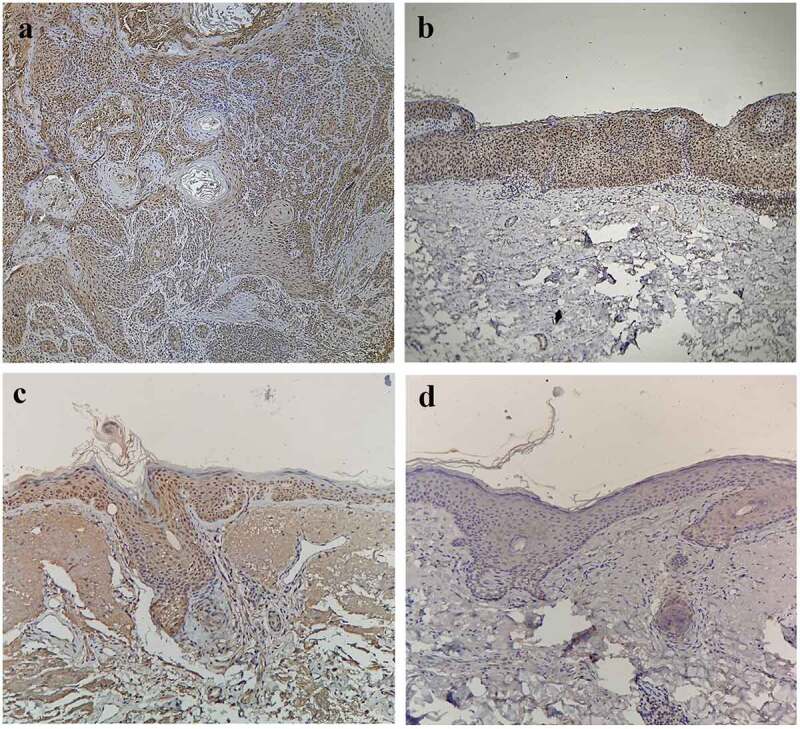
Expressions of Ki-67 in CSCC, BD, AK, and normal skin. A) Positive expression of Ki-67 in CSCC (SP, ×200), Positive(+++). (b) Positive expression of Ki-67 in BD (SP, ×200), Positive(+++). (c) Positive expression of Ki-67 in AK (SP, ×200), Positive(++). (d) Negative expression of Ki-67 in normal skin (SP, ×200)

In the CSCC group, there were a total of 28 cases (93.33%) of positive expression (Figure 2a), dominated by strong positive expression. In the BD group, there were a total of 23 cases (76.67%) of positive expression (Figure 2b), dominated by strong positive expression. In the AK group, there were a total of 18 cases (60.00%) of positive expression (Figure 2c), dominated by moderate positive expression. In the normal skin group, the Ki-67 expression was negative in all cases (Figure 2d), so this group was excluded from the statistical analysis.

It can be seen that the positive expression rate of Ki-67 had statistically significant differences among the CSCC group, BD group, and AK group (χ^2^ = 5.57, *P* < 0.05). Furthermore, it was found through partition of chi-square that the positive expression rate of Ki-67 in the CSCC group was higher than that in the AK group, with a statistically significant difference (χ^2^ = 9.32, *P* < 0.01), while it had no statistically significant difference between the CSCC group and the BD group (χ^2^ = 2.09, *P* > 0.05), and between the BD group and the AK group (χ^2^ = 1.93, *P* > 0.05). Besides, the Ki-67 expression had statistically significant differences among the CSCC group, BD group, and AK group in the Kruskal-Wallis *H* test (*H* = 33.87, *P* < 0.05). The results of pairwise comparison *via* the Nemenyi test revealed that the difference in the Ki-67 expression was statistically significant between the CSCC group and the AK group (*P* < 0.05), but it was not statistically significant between the CSCC group and the BD group and between the BD group and the AK group (*P* > 0.05), as shown in Table 1.

### Correlations between mTOR positive expression and clinicopathological characteristics of CSCC

In the CSCC group (n = 30), the positive expression rate of mTOR was relatively higher in patients with lesion onset at the exposure site, the course of disease ≥2 years, and moderate-poor differentiation (*P* < 0.05), but it had no statistically significant association with gender, age, and tumor diameter. With the mTOR expression as the binary (negative = 0, positive = 1) dependent variable and the factors in Table 2 as independent variables, logistic regression analysis was conducted. The results manifested that after confounding factors were controlled, the pathogenic site, course of disease, and differentiation degree were independent influencing factors for the positive expression of mTOR (*P* < 0.05), as shown in Table 3.

**Table 2.** Univariate analysis results of correlations between mTOR positive expression and clinicopathological characteristics of CSCC

Factor	n	mTOR positive expression	*OR*	95% CI	*P*
Gender			1.327	0.782 ~ 2.256	0.293
Female	13(43.33)	9(69.23)			
Male	17(56.67)	13(76.47)			
Age			1.721	0.917 ~ 2.634	0.082
<60 Y	11(36.67)	6(54.55)			
≥60 Y	19(63.33)	16(84.21)			
Pathogenic site			2.514	1.018 ~ 5.843	0.012
Exposure site	23(76.67)	20(86.96)			
Nonexposure site	7(23.33)	2(28.57)			
Course of disease			0.346	0.275 ~ 0.841	0.009
<2 years	8(26.67)	3(37.50)			
≥2 years	22(73.33)	19(86.36)			
Diameter of tumor			1.93	1.026 ~ 3.201	0.063
<1 cm	14(46.67)	8(57.14)			
≥1 cm	16(53.33)	14(87.50)			
Differentiation degree			2.715	1.059 ~ 6.973	0.027
High	12(40.00)	6(50.00)			
Moderate-poor	18(60.00)	16(88.89)

**Table 3.** Logistic regression analysis results of influencing factors for mTOR positive expression in CSCC

Influencing factor	OR	95%CI	*P*
Pathogenic site	1.189	1.028 ~ 1.381	0.021
Course of disease	2.059	1.036 ~ 4.087	0.043
Differentiation degree	1.325	1.169 ~ 1.512	0.001

### Correlations between mTOR and Ki-67 expressions in CSCC, BD, AK, and normal skin

The results of Spearman’s correlation analysis revealed that the expression levels of mTOR in CSCC, BD, and AK were positively correlated with the expression level of Ki-67 (*r* = 0.827, *P* < 0.01, *r* = 0.608, *P* < 0.01, *r* = 0.368, *P* = 0.045), when the distribution of mTOR and Ki67 positive cells did not overlap in the tumor.

## DISCUSSION

Skin is the largest organ of the human body, accounting for about 20% of the total body weight, which acts as a dynamic, mechanical, and physical defense barrier of the human body against external damage, such as ultraviolet radiation, chemical substances, mechanical irritation, and microbial infection [9]. Skin is more susceptible to damage and stimulation than solid visceral organs, leading to gene mutations and malignant tumors. CSCC, a kind of nonmelanoma skin cancer with an incidence rate second only to that of basal cell carcinoma, accounts for 20% of all skin malignancies [10]. It is reported that the incidence rate of CSCC is increasing year by year [11,12]. At the same time, it has also been found that phosphatidylinositol 3-kinase (PI3K) will be activated in human keratinocytes exposed to long- and medium-wave ultraviolet and the expression of phosphorylated ribosomal protein S6 kinase 1 (S6K1) in human keratinocytes can be suppressed by rapamycin [13], suggesting that the mTOR signaling pathway may play a role in the occurrence of human skin epithelial tumors.

MTOR is an atypical serine/threonine protein kinase, widely present in a variety of eukaryotic cells. It is an evolutionarily conserved member of the PI3K superfamily, which works through two different signaling complexes, mTOR complex 1 (mTORC1) and mTORC2. MTORC1 is sensitive to rapamycin, growth factors, energy (adenosine triphosphate), nutrients (amino acids), oxidative stress, and DNA damage, which regulates cell growth and proliferation through controlling the protein, lipid, and nucleotide synthesis and also regulates lysosomal biogenesis *via* mediating the phosphorylation of S6K1 and eukaryotic initiation factor 4E. MTORC2 is only sensitive to growth factors and long-term (>24 h) exposure to rapamycin, which regulates cell survival and cytoskeletal tissue formation partially through controlling the phosphorylation of protein kinase B, glucocorticoid-induced kinase 1, protein kinase C α, focal adhesion protein, and the activity of guanosine triphosphate. The function of the mTOR complex is yet to be revealed, but current studies have shown that mTOR is a key player in the regulation on cell growth, proliferation, differentiation, survival, autophagy and motility, angiogenesis, and lymphangiogenesis [14–16]. Moreover, research has shown that mTOR was involved in the occurrence of inflammatory skin diseases such as psoriasis and acne [17,18] and the mTOR signaling pathway participates in the occurrence of malignancies in multiple systems. The research on the pathogenesis of mTOR in squamous cell carcinoma mainly focuses on the head-neck squamous cell carcinoma [19–21], but less on CSCC. Meanwhile, the correlation between mTOR and proliferation degree of CSCC and whether there is a difference in the mTOR expression in different skin diseases with squamous cell differentiation abnormality are less reported in domestic and international.

AK, BD, and CSCC are three kinds of skin diseases with squamous cell differentiation abnormality, whose grade of malignancy is gradually increasing in turn. Although mTORC1 can be directly activated by phosphorylation of mTOR site Ser2448, detection of the mTOR positive expression rate can reflect the activation state of the mTOR pathway. Therefore, in this study, immunohistochemical staining was used to detect the expressions of mTOR and Ki-67 in skin tissues of AK, BD, and CSCC, in order to analyze the effect of mTOR on the proliferation of CSCC cells. In the present study, it was found that the positive expression rate of mTOR in the BD group and AK group was higher than that in the normal skin group, indicating that mTOR may be involved in the occurrence of squamous cell carcinoma. The positive expression rate of mTOR in the CSCC group was higher than that in the BD group and AK group, but it had no difference between the BD group and AK group, suggesting that mTOR may be related to the proliferation degree of CSCC and involved in tumor invasion. Moreover, the results showed that the positive expression of mTOR was associated with the pathogenic site, course of disease, and differentiation degree, but it had no correlation with gender, age, and tumor size. The positive expression of mTOR was high in CSCC tissues at the exposure site and with a long course of disease, suggesting that long-term ultraviolet damage may be involved in the onset of CSCC. As an essential enzyme for DNA replication and synthesis into mRNA, Ki-67 can reflect the cell proliferative activity and cycle more accurately, which is currently the most widely used index for assessing the cell proliferation status. In this study, the results of the correlation analysis on mTOR and Ki-67 expressions in CSCC, BD, and AK revealed that the expression level of mTOR was positively correlated with that of Ki-67 in CSCC, BD, and AK, demonstrating that mTOR is related to the proliferation degree of CSCC, and the higher the proliferation level of squamous cells, the higher the positive expression level of mTOR. So we demonstrated that long-term UV exposure could stimulate the activation of the mTOR signaling pathway and promote the proliferation of squamous cells, thus leading to the occurrence of CSCC. As confirmed in the study on laryngeal cancer by García-Carracedo *et al*. [22], mTOR can serve as an index reflecting the proliferation status of CSCC.

Currently, more and more mTOR inhibitors have been applied in the clinical treatment of malignancies, such as lung cancer, laryngeal cancer, and pancreatic cancer [23,24]. Some drugs targeting the mTOR signaling pathway have also been used for CSCC patients, which are now in phase I/II clinical trials [25]. In addition, the effectiveness of mTOR inhibitors (rapamycin, sirolimus, and everolimus) for post-transplantation CSCC has also been reported [26]. With the constant deepening of research, the role of the mTOR signaling pathway in the pathogenesis of CSCC has been gradually clarified and anticancer drugs targeting the mTOR signaling pathway may become new targeted drugs for CSCC. In the present study, the expressions of mTOR and Ki-67 in CSCC, BD, and AK tissues were detected and analyzed. It was confirmed that mTOR may be involved in the onset of CSCC and mTOR is related to the proliferation degree of CSCC and can act as an index, reflecting the proliferation status of CSCC.

However, due to a small sample size and a single detection method in this study, the role of mTOR in the growth, proliferation, invasion, and metastasis of CSCC cells and the influence of ultraviolet radiation on mTOR expression remain to be deeply explored in the future multicenter large-sample research.

## CONCLUSION

In this study, the expressions of mTOR and Ki-67 in CSCC, BD, and AK tissues were detected and analyzed. It was confirmed that long-term uviolize could stimulate the activation of the mTOR signaling pathway and promote the proliferation of squamous cells, thus leading to the occurrence of CSCC. mTOR can act as an index reflecting the proliferation status of CSCC.

## Research highlights

1. The positive expression rate of mTOR in CSCC was higher than that in BD/AK tissues.
2. The positive expression rate of Ki-67 in CSCC was higher than that in AK tissues.
3. The expression level of mTOR in CSCC was positively correlated with that of Ki-67.
4. The activation of the mTOR signaling pathway promotes the proliferation of CSCC.
